# Dataset on transcriptomic profiling of cholestatic liver injury induced by food additives and a cosmetic ingredient

**DOI:** 10.1016/j.dib.2021.107373

**Published:** 2021-09-17

**Authors:** Eva Gijbels, Lindsey Devisscher, Mathieu Vinken

**Affiliations:** a Department of *In Vitro* Toxicology and Dermato-Cosmetology, Vrije Universiteit Brussel, Laarbeeklaan 103, Brussels 1090, Belgium; b Gut-Liver Immunopharmacology Unit, Basic and Applied Medical Sciences, Liver Research Center Ghent, Faculty of Medicine and Health Sciences, Ghent University, Corneel Heymanslaan 10, Ghent 9000, Belgium

**Keywords:** Chemical-induced cholestasis, Cosmetic ingredient, Food additive, Microarray, Transcriptomics

## Abstract

The provided dataset describes the differential gene expression profile of human hepatoma HepaRG cells cultured in monolayer configuration upon treatment with chemical compounds with cholestatic potential, including food additives sunset yellow and tartrazine and cosmetic ingredient triclosan, while being exposed to a highly concentrated bile acid mixture. Whole genome microarray Affymetrix Human U133 plus 2.0 was used to obtain the raw data followed by normalization, summarization and background adjustments by means of Robust Multichip Average Express software. Raw data of the different conditions were included as .CEL files in the Gene Expression Omnibus with accession number GSE169072. These data may serve as the basis for further refinement studies to establish an adequate transcriptomic signature of chemical-induced cholestasis fit-for-purpose in screening the cholestatic liability of different types of chemical compounds.

## Specifications Table

**Table:** 

Subject	Bioinformatics, hepatology and toxicology.
Specific subject area	Cholestatic liver injury
Type of data	Raw Data
How data were acquired	Affymetrix GeneChip Human Genome U133 plus 2.0 array (ThermoFisher, Belgium) Ingenuity Pathway Analysis (IPA) (Qiagen, Belgium) Transcriptome Analysis Console (TAC) (ThermoFisher, Belgium)
Data format	Raw (.CEL), normalized and analyzed
Parameters for data collection	Cryopreserved differentiated human hepatoma HepaRG cells (Biopredic International, France) were cultured according to the manufacturer's instructions (Biopredic International, France). HepaRG cells were exposed to 13 mM acetaminophen (APAP), 10 mM sunset yellow (SUN), 50 mM tartrazine (TART) and 0.050 mM triclosan (TRI). Simultaneously, HepaRG cells were co-exposed to a bile acid (BA) mixture, which is 50 × concentrated and consists of 5 BAs, including 66 µM glycochenodeoxycholic acid, 20 µM deoxycholic acid, 19.5 µM chenodeoxycholic acid, 19 µM glycodeoxycholic acid, and 17.5 µM glycocholic acid. Both the 50 × concentrated BA mixture and the tested compounds were included in the cell culture medium of HepaRG cells from day 7 after seeding. HepaRG were routinely exposed for 72 h with daily renewal of the cell culture medium, including the 50 × concentrated BA mixture and tested chemicals. Dimethyl sulfoxide (DMSO) treated HepaRG cells served as control. All conditions contained the same final DMSO concentration of 0.25%. All chemicals were purchased from Sigma Aldrich, Belgium.
Description of data collection	Total RNA extraction was performed on HepaRG cell culture samples after 72 h exposure to APAP, SUN, TART and TRI in the presence of a 50 × concentrated BA mixture (*i.e.* APAP + BA, SUN + BA, TART + BA and TRI + BA), as well as from HepaRG cells solely exposed to the 50 × concentrated BA mixture (*i.e.* BA) and from the respective vehicle control (CTL). 3 different HepaRG batches were used throughout the study (*n* = 3). Quantification and purity of the isolated RNA were evaluated *via* spectrophotometric analysis, namely the Nanodrop spectrophotometer (ThermoFisher Scientific, Belgium). Whole genome expression analysis was performed using microarray technologies (Affymetrix, Germany).
Data source location	Department of *In Vitro* Toxicology and Dermato-Cosmetology, Vrije Universiteit Brussel, Jette, Belgium.
Data accessibility	Raw data is available at the Gene Expression Omnibus (GEO) from The National Center for Biotechnology Information (NCBI) with access number GSE169072. https://www.ncbi.nlm.nih.gov/geo/query/acc.cgi?acc=GSE169072
Related research article	E. Gijbels, L. Devisscher, M. Vinken, Testing *in vitro* tools for the prediction of cholestatic liver injury induced by non-pharmaceutical chemicals, Food Chem Toxicol (2021) doi:10.1016/j.fct.2021.112165[1]

## Value of the Data

• This data reflects a genome-wide gene expression signature of non-pharmaceuticals, including food additives and a cosmetic ingredient, with a presumed cholestatic liability.
• The data may assist further research in the development of *in vitro* tools applicable for assessing the cholestatic liability of both pharmaceutical as non-pharmaceutical compounds, while identifying novel mechanisms underlying chemical-induced cholestasis.
• The data supports possible follow-up experiments that refine the established transcriptomic signature of cholestasis, as foundation for an improved technique that accurately predicts different types of chemical-induced cholestasis.

## 1 Data Description

**Fig. 1:**
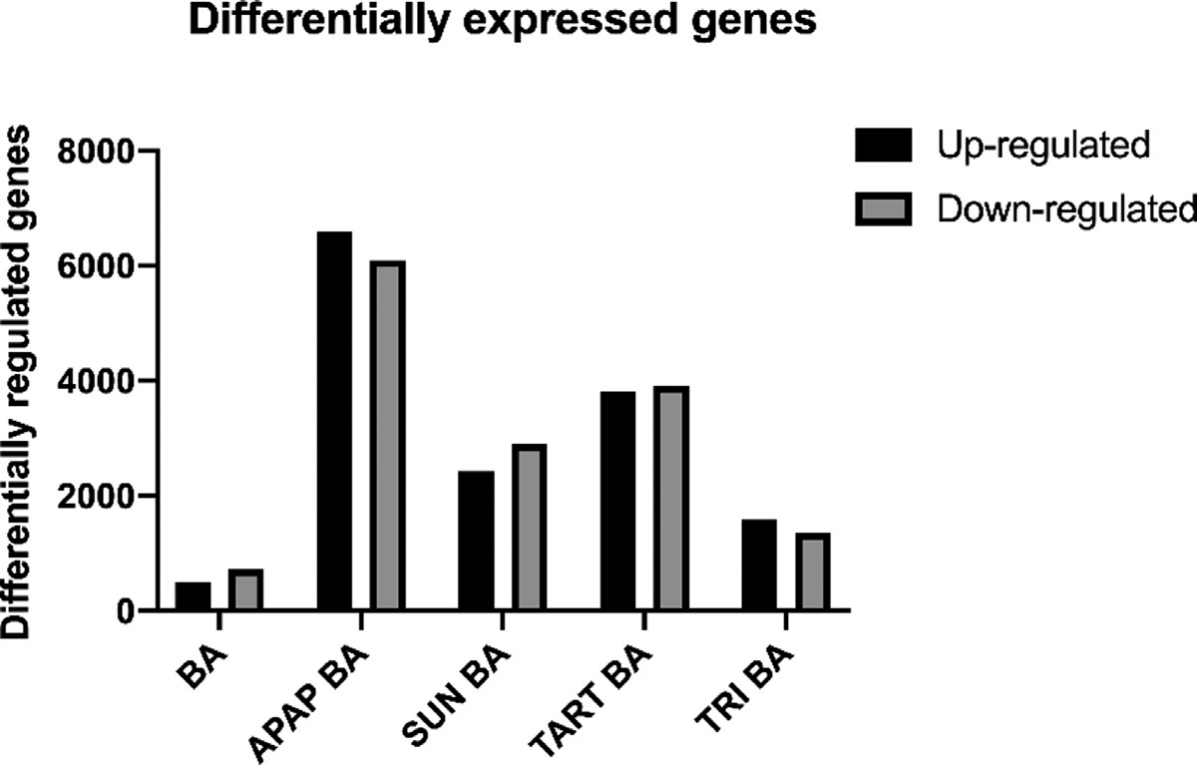
Number of differentially expressed genes. The absolute number of significantly up- and down-regulated genes after exposing the HepaRG cells to BA, APAP BA, SUN BA, TART BA and TRI BA compared to the respective control. [Cut-off fold change of [−1.5; 1.5], *p*-value <0.05 calculated *via* ANOVA and False Discovery Rate *p*-value <0.1 with Benjamini-Hochberg correction]. Transcriptome Analysis Console Software was used.

**Fig. 2:**
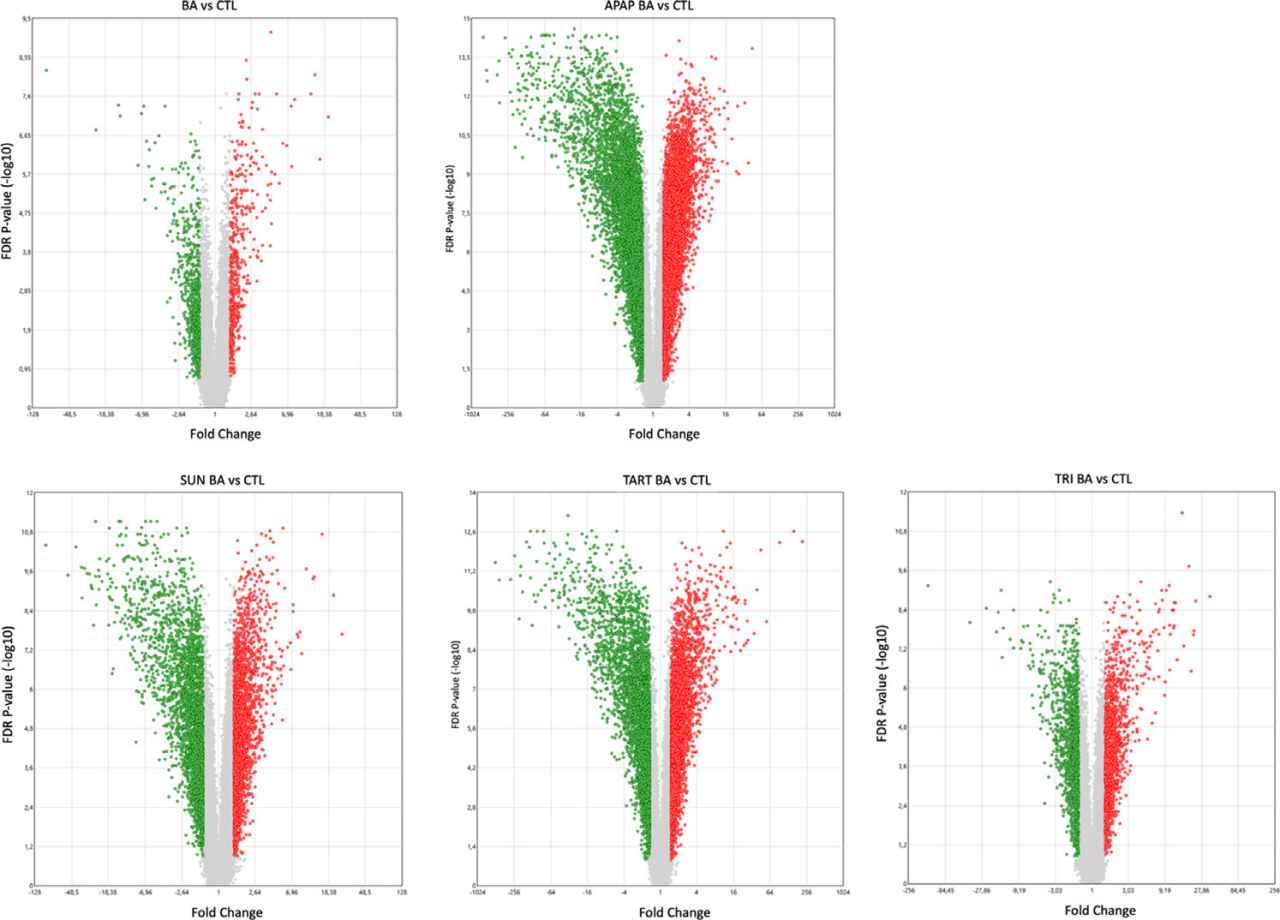
Volcano plots representing the significantly modulated probe sets after exposure to BA, APAP BA, SUN BA, TART BA and TRI BA *versus* the respective control. [Only significantly modulated genes are visualized with a cut-off fold change of [−1.5; 1.5] and False Discovery Rate *p*-value <0.05 calculated *via* ANOVA and Benjamini-Hochberg correction] [Green = down-regulated, red = up-regulated]. Transcriptome Analysis Console Software was used. (For interpretation of the references to color in this figure legend, the reader is referred to the web version of this article.)

**Fig. 3:**
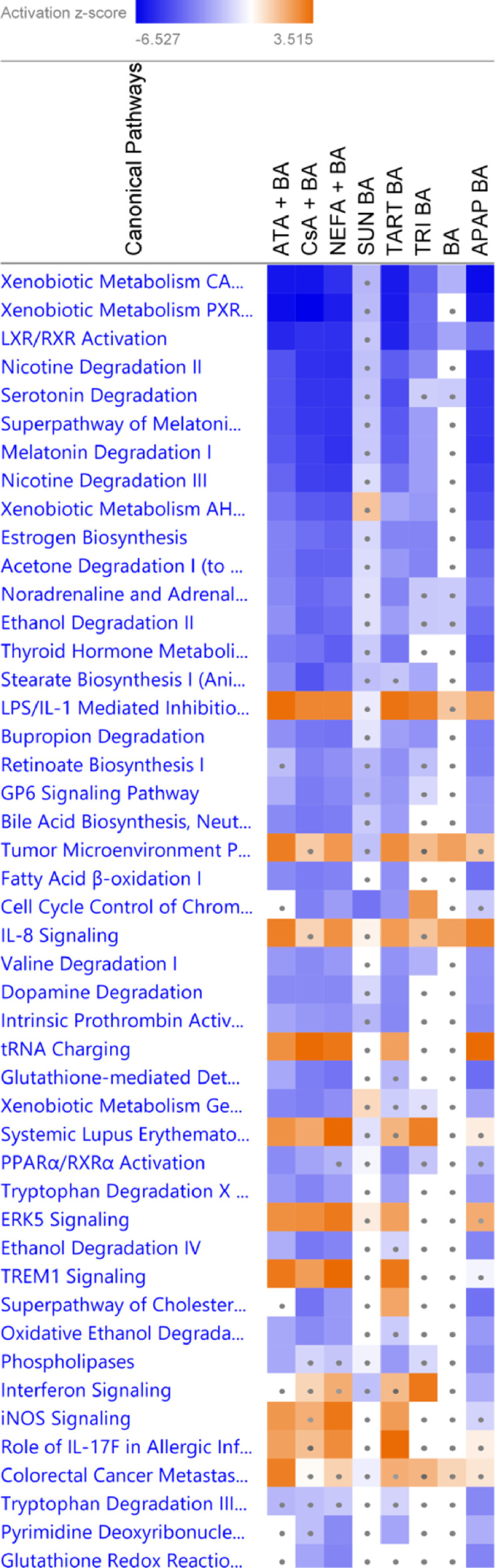
Canonical pathway analysis of the altered gene expression levels after exposure to ATA + BA, CsA + BA, NEFA + BA, SUN + BA, TART + BA, TRI + BA, BA and APAP + BA compared to the respective control. [*Z*-scores were used to predict the activation scores with a cut-off absolute value of 2] [Blue = predicted deactivated, orange= predicted activated, canonical pathways with absolute values lower than 2 are marked with dots]. Ingenuity Pathway Analysis was used. (For interpretation of the references to color in this figure legend, the reader is referred to the web version of this article.)

A total of 3 different batches of HepaRG cells were exposed to 3 non-pharmaceutical compounds, namely the food additives SUN (10 mM) and TART (50 mM) and cosmetic ingredient TRI (0.050 mM), which were believed to bear a cholestatic risk [2,3], in the presence of a 50 × concentrated BA mixture (*i.e.* datasets SUNSET + BA 1-3; TART + BA 1-3 and TRI + BA 1-3). Next to these cholestatic compounds, the non-cholestatic hepatotoxic compound APAP was included in the transcriptomic analysis to verify the specificity of the obtained profile of cholestatic liver injury. HepaRG cells were exposed to the cytotoxic concentration that achieves 50% viability (IC_50_) of APAP, being 13 mM, in the presence of the 50 × concentrated BA mixture (*i.e.* datasets APAP + BA 1-3). HepaRG cells were also solely exposed to the 50 × concentrated BA mixture to identify BA-induced transcriptional changes (*i.e.* datasets BA 1-3). The control consisted of HepaRG cells solely exposed to the vehicle (*i.e.* datasets CTL 1-3). After 72 h of exposure, HepaRG cells were sampled, and RNA was isolated. Transcriptomic data were obtained using Affymetrix Human Genome U133 plus 2.0 which, in turn, were processed by the software program Robust Multichip Average (RMA) Express [4]. Gene expression analysis was performed with Transcriptome Analysis Console Software (version 4.0.025, Applied Biosystems) and Ingenuity Pathway Analysis (version 62089861, Qiagen) [5] with a cut-off fold change of ≤−1.5 and ≥1.5, and False Discovery Rate *p*-value <0.05 calculated *via* ANOVA and Benjamini-Hochberg correction. Transcriptomic data generated by the different conditions were visualized by presenting the absolute number of significantly modulated genes (Fig. 1) and volcano plots (Fig. 2). The top 30 differentially expressed genes of each condition were presented in Table 1. Potentially interesting canonical pathways related to cholestasis were introduced in Fig. 3 and compared to previously published datasets in HepaRG cells treated with the cholestatic drugs, atazanavir (ATA, 60 µM), cyclosporin A (CsA, 20 µM) and nefazodone (NEFA, 30 µM) in the presence of a 50 × concentrated BA mixture [6].

**Table 1:** Top 30 differentially expressed genes. The top 30 significantly up- and down-regulated genes after exposing the HepaRG cells to BA, APAP BA, SUN BA, TART BA and TRI BA compared to the respective control. [Cut-off fold change of [−1.5; 1.5], *p*-value <0.05 calculated *via* ANOVA and False Discovery Rate *p*-value <0.1 with Benjamini-Hochberg correction]. Transcriptome Analysis Console Software was used.

Top 30 differentially regulated genes	BA	APAP + BA	SUN + BA	TART + BA	TRI + BA
Upregulated genes	SLC51A, ABCB11, SLC51B, ITIH3, FETUB, RTP3, DHRS9, FNDC5, GPLD1, NEFL, PEG10, TCEA2, FABP4, PAGE4, PTGFR, KRT222, MFSD2A, MMP10, AOC3, CXCL11, TBX3, PALMD, PDE7B, SPX, IGF1, ABCB4, BTBD11, GNA13, TPM1, NCF2	TAC1, UNC5B, PTX3, MMP10, ATF3, CCDC85B, SLC7A11, ARG2, ARL4C, UPP1, PPM1L, SLC4A7, ASNS, LCN2, QPCT, GOLGA2P10, DDIT3, NEBL, ATP8A1, IGFBP3, STON2, SLC1A4, IFRD1, SLC7A11, SLFN5, GPC5, LMO4, TSLP, KITLG, SHISA2	CYP1A1, QPCT, CYP1A2, AKR1C1, SLC51B, SLC51A, OLMALINC, TUBA1A, ATP8B5P, NEFL, BTBD11, AQP4, PEG10, ABCB11, FHL1, DCLK1, RASGRP1, HTATIP2, DCLK1, TSGA10, WDR78, KRT222, ABCG1, INSC, RTP3, PNLIPRP3, MYOT, TNFSF11, LAMA1, FHL1	TNFAIP6, TAC1, PNLIPRP3, IL1RL1, PTX3, MMP10, PTGS2, EBI3, PI3, CCL2, MMP1, LCN2, SLC5A3, SLC39A8, CALB1, IL6, AKR1C1, TMEM171, ATP8B5P, DNER, SLC5A3, SERPINE2, TFPI2, ARG2, MEDAG, CXCL8, WFDC21P, ZC3H12A, TNC, EVI2B	IFI44L, CPMK2, SLC51A, CXCL11, HSPA6, MX1, IFITM1, IFI27, RSAD2, DHRS9, SLC51B, MX2, OAS2, MMP10, ITIH3, IFI6, IFIT1, LAMP3, ACBC11, OAS1, OASL, EPSTI1, ISG15, IFIT3, IFI44, HERC6, XAF1, HSPA6, FETUB, XAF1

Downregulated genes	CYP7A1, ALDOB, ADH4, CRP, CYP2E1, C9, CFHR4, C4BPA, ADH1C, SPP2, PCK1, ASCL1, CFHR3, PGLYRP2, SULT1E1, CPS1, PPP1R1A, AMDHD1, SLC22A1, INHBA, ETNPPL, PKLR, KMO, AGXT2, SULT2A1, AKR1D1, VNN1, OTC, HAO2, MRC1	ADH1B, UGT2B4, CXCL13, CYP2C8, FABP1, GBA3, ADH1B, HSD17B6, ADH4, FGA, SULT2A1, ADH1A, ACSM2A, GBA3, ALDOB, SERPINA7, UGT2A3, TM4SF4, UGT2B15, MMTP, HNMT, ARG1, HSD17B6, AFM, AHSG, CYP2E1, KCNJ16, VNN1, CYP7A1, HMGCS2	ADH4, CYP7A1, DPT, COL3A1, ALDOB, PRKAR2B, CYP2E1, DCN, C9, C1QTNF7, RRM2, MCTP1, CRP, SPC25, CXCL13, DLGAP5, AFM, EGR2, LBP, CFHR4, KI20A, ANLN, CPS1, CDK1, AHSG, ALDH8A1, FAM111B, CEP55, KIF14, PLAC8	ALDOB, ACSM2A, FABP1, AHSG, ADH4, AFM, GBA3, ARG1, CYP2C8, SLC2A2, CYP7A1, CYP2C9, GPR88, F9, MTTP, SLC22A7, ANGPTL3, SERPINA7, SULT2A1, F13B, CYP2E1, G6PC, OTC, LEAP2, ADH1A, HMGCS2, CFHR4, CPS1, SLC22A1, CYP4A11	CYP7A1, ALDOB, ADH4, CYP2E1, C9, ADH1C, PCK1, CPS1, CFHR4, SULT1E1, CPS1, OTC, ADH1B, MRC1, SULT2A1, ASCL1, SLC22A1, PGLYRP2, C4BPA, HAO2, SPP2, ETNPPL, GLYAT, AKR1D1, ACOT12, HAO2, ACSM3, PIPOX, PKLR, PPP1R1A

## 2 Experimental Design, Materials and Methods

Cryopreserved differentiated HepaRG cells, including the required culturing reagents, were all purchased from Biopredic International, France. HepaRG cells were thawed and seeded in basal hepatic medium enriched with HepaRG Thawing/Plating/General Purpose Medium Supplement with antibiotics following manufacturer's instructions. HepaRG cells were seeded onto 24 well plates at a concentration of 0.48 × 10^6^ cells/well coated with rat tail collagen (0.1 mg/ml) (Corning, Sigma Aldrich, Belgium). Afterwards, the HepaRG cells were allowed to attach for about 24 h and subsequently refreshed every 2, 3 days with basal hepatic medium enriched with Maintenance/Metabolism Medium Supplement with antibiotics. After 7 days of cultivation, HepaRG cells were exposed to APAP (13 mM), SUN (10 mM), TART (50 mM) and TRI (0.050 mM) (Sigma Aldrich, Belgium) with a 50 × concentrated BA mixture (*i.e.*66 µM glycochenodeoxycholic acid, 20 µM deoxycholic acid, 19.5 µM chenodeoxycholic acid, 19 µM glycodeoxycholic acid, and 17.5 µM glycocholic acid (Sigma Aldrich, Belgium)) added to the cell culture medium. Stock solutions from TRI and the BAs were made in DMSO. The final incubation solutions were prepared *ex tempore* by diluting the stock solution with basal hepatic cell medium enriched with Serum-free Induction Medium Supplement with antibiotics (Biopredic International, France). Final solutions of APAP, SUN and TART were directly prepared in the induction serum-free medium. All conditions contained a final DMSO concentration of 0.25% v/v. HepaRG cells were exposed for 72 h to the tested compounds and the BA mixture. Afterwards, cell culture medium was aspirated, and cells were lysed by means of a lysis buffer (lysis solution with 1/100 β-mercaptoethanol, Qiagen, Belgium). Total RNA extraction was performed with the RNeasy Mini Kit (Qiagen, Belgium) according to manufacturer's instructions. Subsequently, purity and quantification of the acquired and isolated RNA were determined *via* spectrophotometric analysis by means of a NanoDrop® ND-100 Spectrophotometer (ThermoFisher Scientific, Belgium). A cut-off ratio of [1.8–2.1] for the absorption at 260/280 nm was respected during the purity assessment.

Microarray technologies from Affymetrix (Germany) were used to perform whole genome expression, more specifically by means of the GeneChip 3′IVT Express Kit and in agreement with previous studies from Gijbels et al. [6,7]. Herein, 100 ng total RNA per sample was used to synthesize first and second strand of cDNA. Second strand cDNA was, in turn, used as template to produce and amplify biotin labelled complementary RNA using the T7 RNA polymerase. Amplified labelled RNA was purified with magnetic beads, after which the acquired RNA yield was assessed, and fragmented by divalent cations and elevated temperature. Subsequently, 12.5 µg of the fragmented amplified RNA was hybridized to microarray chips (Affymetrix Human genome U133 plus 2.0 GeneChip, Germany). The chips were then placed in a GeneChip Hybridization Oven 645 (Affymetrix, Germany), after which the arrays were washed with GeneChip Fluidics Station 450 (Affymetrix, Germany) and stained with Affymetrix HWS kit. Next, the Affymetrix GeneChip Scanner 3000 7G scanned the stained arrays. Hybridization controls and normalization quality controls, such as average and background intensities, noise, raw Q-values, scaling factors and present calls were all done with the Affymetrix GCOS and RMA Express software, respectively, and were within the acceptable limits of employed microarray chips.

## CRediT Author Statement

**Eva Gijbels:** Conceptualization, Formal analysis, Funding acquisition, Writing – original draft; **Lindsey Devisscher:** Writing – review & editing; **Mathieu Vinken:** Conceptualization, Writing – original draft, Writing – review & editing, Funding acquisition, Supervision.

## Declaration of Competing Interest

The authors declare that they have no known competing financial interests or personal relationships which have, or could be perceived to have, influenced the work reported in this article.
